# Generation of 3D Skin Equivalents Fully Reconstituted from Human Induced Pluripotent Stem Cells (iPSCs)

**DOI:** 10.1371/journal.pone.0077673

**Published:** 2013-10-11

**Authors:** Munenari Itoh, Noriko Umegaki-Arao, Zongyou Guo, Liang Liu, Claire A. Higgins, Angela M. Christiano

**Affiliations:** 1 Department of Dermatology, Columbia University, College of Physicians & Surgeons, New York, New York, United States of America; 2 Department of Genetics & Development, Columbia University, College of Physicians & Surgeons, New York, New York, United States of America; University of Maryland School of Medicine, United States of America

## Abstract

Recent generation of patient-specific induced pluripotent stem cells (PS-iPSCs) provides significant advantages for cell- and gene-based therapy. Establishment of iPSC-based therapy for skin diseases requires efficient methodology for differentiating iPSCs into both keratinocytes and fibroblasts, the major cellular components of the skin, as well as the reconstruction of skin structures using these iPSC-derived skin components. We previously reported generation of keratinocytes from human iPSCs for use in the treatment of recessive dystrophic epidermolysis bullosa (RDEB) caused by mutations in the *COL7A1* gene. Here, we developed a protocol for differentiating iPSCs into dermal fibroblasts, which also produce type VII collagen and therefore also have the potential to treat RDEB. Moreover, we generated *in vitro* 3D skin equivalents composed exclusively human iPSC-derived keratinocytes and fibroblasts for disease models and regenerative therapies for skin diseases, first demonstrating that iPSCs can provide the basis for modeling a human organ derived entirely from two different types of iPSC-derived cells.

## Introduction

 Induced pluripotent stem cells (iPSCs) are stem cells generated from individual somatic cells by exogenous expression of several transcription factors to initiate the reprogramming process [1]. In recent years, patient-specific iPSCs (PS-iPSCs) have been derived from patients with several human diseases to investigate unknown disease mechanisms and perform pre-clinical testing in various models [2]. Autologous PS-iPSCs have the potential to provide an unlimited source of cells for gene and cell therapies for specific human diseases, since they are believed to have unlimited proliferative capacity and extensive differentiation capability into a wide range of cell types. 

Recessive dystrophic epidermolysis bullosa (RDEB) is an inherited blistering disorder caused by mutations in the *COL7A1* gene encoding type VII collagen, the major component of anchoring fibrils at the basement membrane zone (BMZ) at the epidermal-dermal junction of the skin. Since anchoring fibrils provide functional integrity to the skin, defective anchoring fibrils caused by lack or deficiency of type VII collagen leads to skin fragility. Therefore, patients with RDEB develop severe skin phenotypes, including repeated skin blistering from minor trauma, as well as mutilating scarring, alopecia, corneal erosions, tooth and nail dystrophy, esophageal strictures, joint contractures, and fusion of fingers and toes [3]. RDEB patients also present an increased risk of developing squamous cell carcinomas (SCCs) in early adulthood as a result of repeated and aberrant wound repair and chronic inflammation. Furthermore, since SCCs in patients with RDEB are highly aggressive and metastatic, SCCs are one of the most life-threatening complications for RDEB patients [4]. 

There is currently no cure for RDEB. One promising strategy for RDEB treatment is to increase the amount of type VII collagen at the epidermal-dermal junction in patients with RDEB to restore skin integrity [5]. Various trials are being pursued using gene-, protein-, drug- and cell-based approaches to address this challenge. Although keratinocytes produce the majority of collagen VII in the skin *in vivo*, several studies using mouse models and mouse/human fibroblasts have shown that dermal fibroblasts also synthesize type VII collagen, suggesting that both cell types are potentially useful as a source of cells for treating RDEB [6–10]. Previous clinical studies have shown that a single intradermal injection of allogeneic fibroblasts led to increased expression of type VII collagen at the epidermal-dermal junction in patients with RDEB [10]. Although donor fibroblasts were undetectable in recipient skin 2 weeks after injection, the newly produced type VII collagen persisted for 3 months, suggesting that cell-based approaches that introduce exogenous type VII collagen-producing cells, such as fibroblasts, might have a therapeutic benefit. 

We previously reported the generation of PS-iPSCs from patients with RDEB and efficient derivation of functional keratinocytes from normal and RDEB PS-iPSCs, thereby demonstrating the utility of PS-iPSCs for establishing cell- and gene-based therapy for RDEB [11]. In contrast, the methodology for generating dermal fibroblasts from ESCs/iPSCs has not yet been fully defined. Therefore, the goal of this study was to establish proof-of-principle for differentiating iPSCs into dermal fibroblasts with high efficiency to provide a new source of cells for RDEB treatment, and further to develop 3D skin equivalents derived exclusively from human iPSCs. 

## Results

### Derivation of Fibroblasts from Human iPSCs

 We first aimed to develop a differentiation protocol to generate dermal fibroblasts from iPSCs in vitro. In previous studies, ESCs have been differentiated into mesodermal lineages using several methods and reagents. Ascorbic acid is well known to induce mesodermal differentiation of ESCs [12,13] and increase collagen synthesis [14], and is widely used to generate mesodermal components from ESCs [15,16]. Moreover, it has been reported that members of the TGFβ family can also enhance not only mesodermal differentiation from stem cells [17–19], but also collagen production in somatic cells, including fibroblasts [20,21]. In particular, TGFβ2 has been reported to involve in epithelial-to-mesenchymal transition in several contexts [22,23]. Other studies also demonstrated that embryoid body formation could lead to spontaneous differentiation of ESCs, and provide fibroblast-like spindle-shaped cells with the capability to maintain ESC pluripotency as feeder cells [24–26]. 

**Figure 1:**
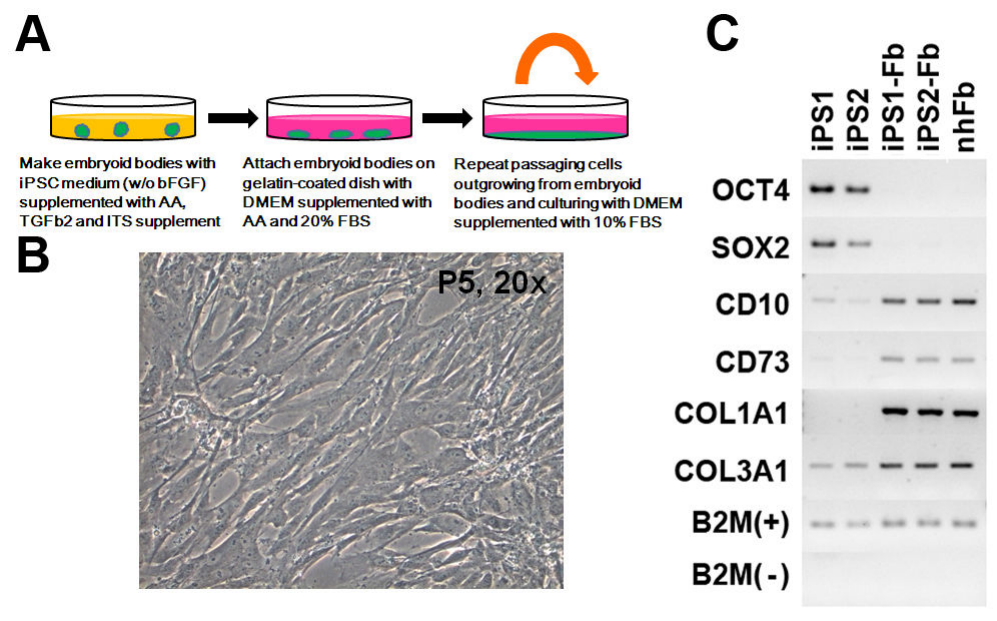
Directed differentiation of iPSCs into fibroblasts. (A) Schematic representation of the differentiation strategy for generating fibroblasts from iPSCs. Cells growing out from attached embryoid bodies made in medium supplemented with AA and TGFβ2 were repeatedly split to obtain a fibroblast-like cell population. (B) Morphology of iPSC-derived fibroblasts. After 5 passages, fibroblast-like spindle-shape cells were observed. (C) Gene expression in iPSC-derived fibroblasts. Expression of stem cell markers (OCT4 and SOX2) disappeared, and markers of fibroblasts (CD10, CD73, type I and III collagen) were increased in iPSC-derived fibroblasts compared with iPSCs.

Based on such studies, we generated embryoid bodies from iPSCs to initiate differentiation, and used medium supplemented with ascorbic acid and TGFβ2 to accelerate mesodermal differentiation to generate fibroblasts from iPSCs (Figure 1A). As a result, we observed fibroblast-like spindle-shaped cells outgrowing from attached embryoid bodies, and relatively uniform population of fibroblast-like cells was obtained after several passages (Figure 1B). 

### Characteristics of iPSC-Derived Fibroblasts

**Figure 2:**
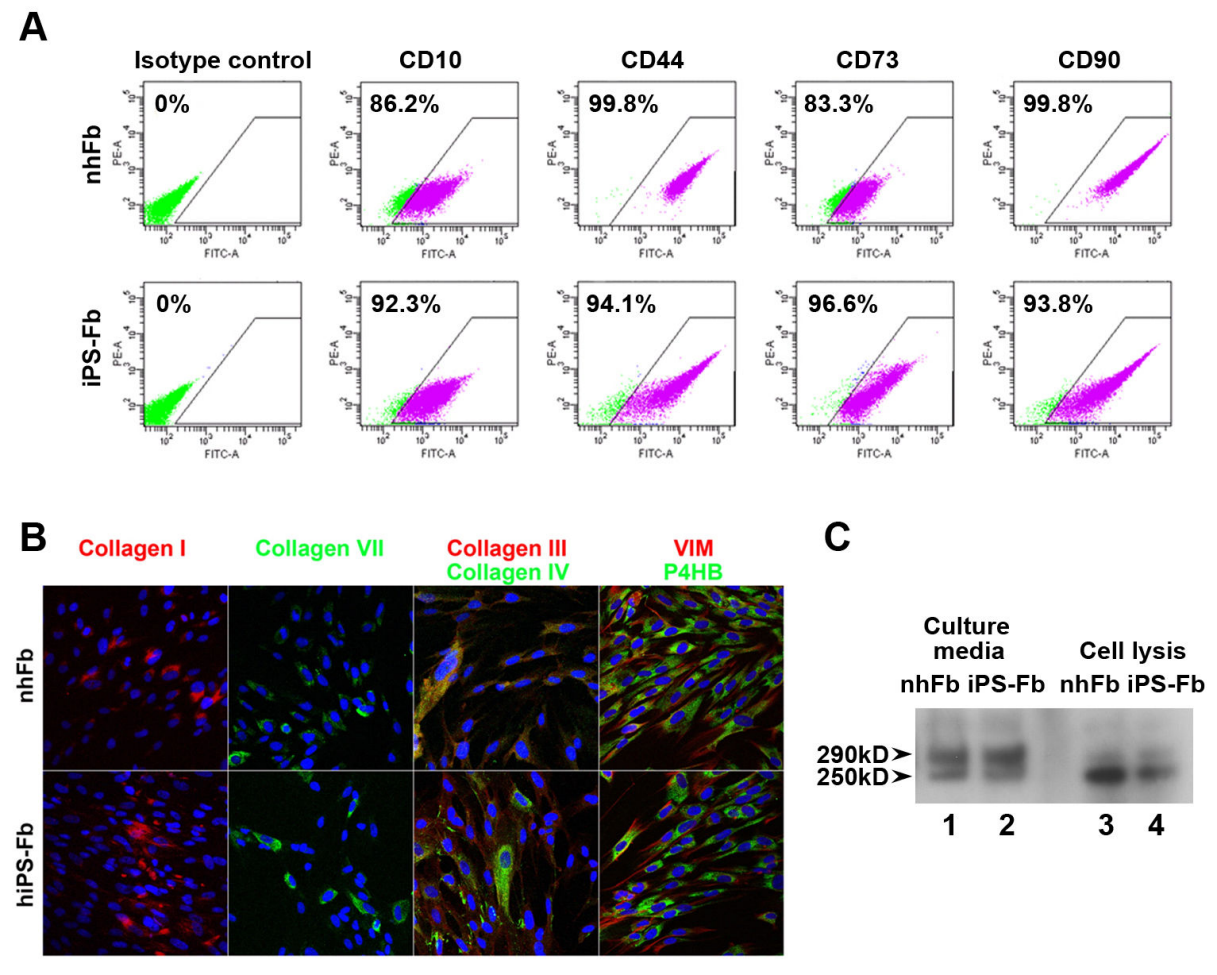
Characteristics of iPSC-derived fibroblasts. (A) Cell surface marker expression in iPSC-derived fibroblasts. FACS analysis showed that the cell surface marker profile of iPSC-derived fibroblasts was very similar to normal human fibroblasts, with over 90% expression of each marker. (B) Protein expression of iPSC-derived fibroblasts. Immunostaining demonstrated that iPSC-derived fibroblasts express fibroblast markers vimentin (VIM) and prolyl-4-hydroxylase beta (P4HB), and produced multiple types of collagen as well as normal human fibroblasts. (C) Detection of mature form of type VII collagen. Western blotting analysis detected 290kD bands in each lane, indicating that type VII collagen was synthesized from iPSC-derived fibroblasts, as well as from normal fibroblasts. The lower band of 250kD may be from either degradation or an immature form of type VII collagen, since it appears to be more abundant in the cells (lanes 3 and 4) than in the culture media (lanes 1 and 2).

 We next examined the characteristics of iPSC-derived cells to define their fibroblast-like properties. We confirmed by RT-PCR that stem cell markers, OCT4 and SOX2, had completely disappeared in iPSC-derived cells after differentiation. In addition, the expression of several markers indicative of mesodermal and fibroblast induction were strongly detected (CD73 and type I collagen) or increased (CD10 and type III collagen. These markers were also weakly expressed in ESC (data not shown)) in iPSC-derived cells (Figure 1C). We compared the expression of fibroblast-associated CD surface markers, CD10, CD44, CD73 and CD90, between normal human fibroblasts and iPSC-derived fibroblasts by FACS. These markers were found in greater than 90% of iPSC-derived fibroblasts (CD10: 93.9±2.8%, CD44: 95.9±2.7%, CD73: 94.1±1.9%, CD90: 96.3±1.9% (n=3)), and their expression patterns in iPSC-derived fibroblasts were consistent with those of dermal fibroblasts (Figure 2A). 

 Moreover, we confirmed by immunostaining that vimentin, a classic marker of the mesodermal lineage, and prolyl-4-hydroxylase beta (P4HB), another marker of fibroblasts, were expressed in iPSC-derived fibroblasts (Figure 2B). P4H, consisting of two alpha and beta subunits (P4HB), is a multifunctional enzyme that belongs to the protein disulfide isomerase family. It plays an essential role in the synthesis of all collagens by fibroblasts, since it is required for hydroxylation of prolyl residues in preprocollagens [27]. Consequently, our data demonstrate that iPSC-derived fibroblasts expressed multiple types of collagens, specifically types I, III, IV and VII, which are hallmarks of normal dermal fibroblasts (Figure 2B). 

### Capability of iPSC-Derived Fibroblasts to Synthesize Type VII Collagen

 To further interrogate the utility of iPSC-derived fibroblasts for the treatment of RDEB, we next asked whether these cells produced and secreted mature type VII collagen, which would be required to reconstitute anchoring fibrils in the skin of patients with RDEB. Western blot analysis detected a 290kD band of type VII collagen in secreted media from iPSC-derived fibroblasts, as well as from normal human fibroblasts (Figure 2C). The lower band may be the result of degradation or an immature form of type VII collagen, because it appears to be more abundant in the cells than in the culture media. This data demonstrated that iPSC-derived dermal fibroblasts have the capability to produce and secrete mature type VII collagen polypeptides. 

### Skin Reconstitution Using iPSC-Derived Fibroblasts

**Figure 3:**
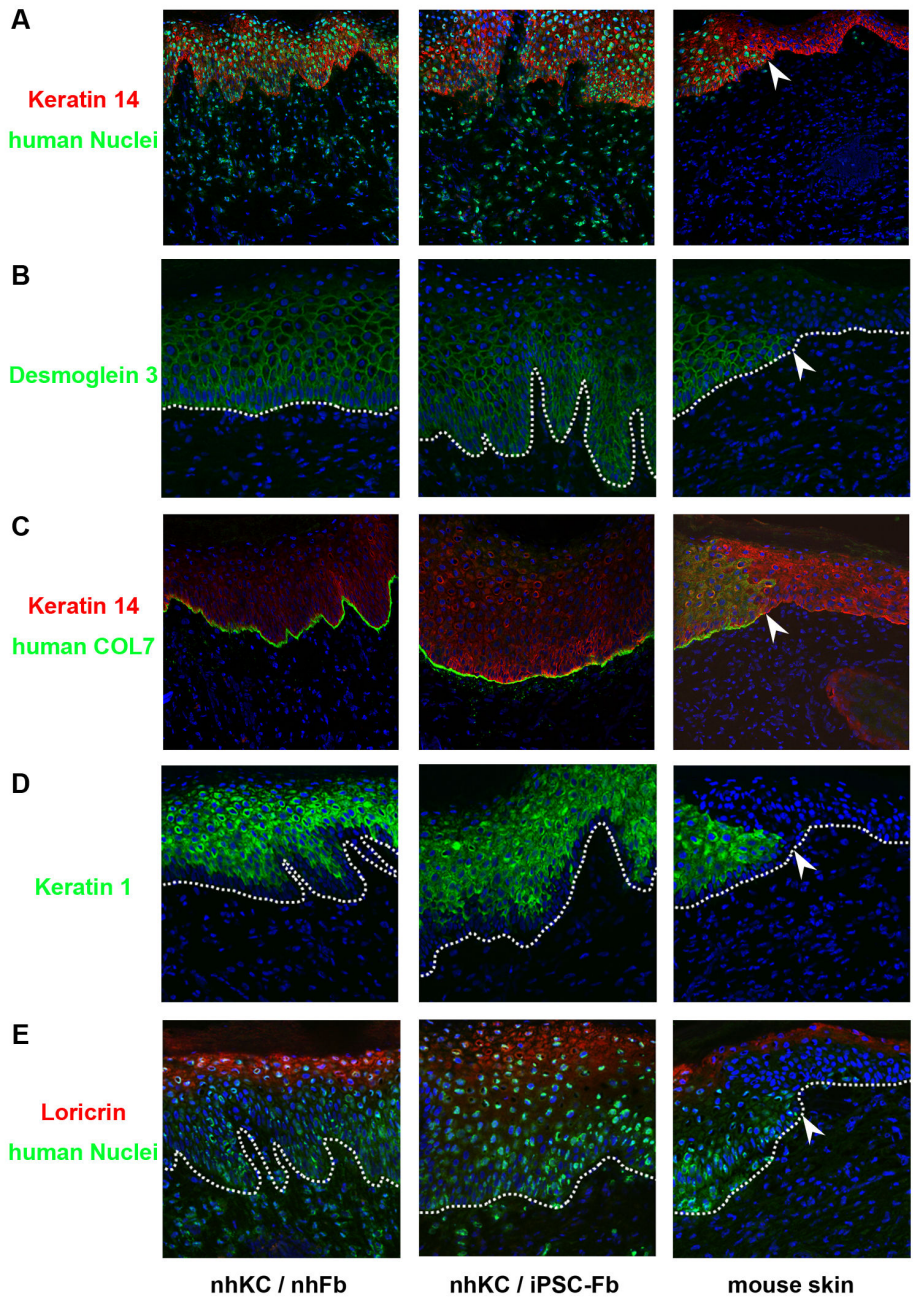
Generation of reconstituted skin using iPSC-derived fibroblasts. (A) iPSC-derived fibroblasts can contribute to forming normal skin structures *in* *vivo*, comparable to normal human fibroblasts. Staining with an anti-human nuclear antibody (green signal) clearly shows the presence of human cells on the back skin of the mice. The white arrow indicates the border of the human cells on the back skin of mouse. Red: keratin 14. Green: human nuclei. Blue: DAPI (both human and mouse nuclei). (B) Desmoglein 3 is expressed though the epidermis. Green: human desmoglein 3 (does not recognize mouse desmoglein 3). Blue: DAPI. Dotted line: BMZ. (C) Human type VII collagen (using LH7.2 antibody which is specific for human type VII collagen) is observed at the BMZ of reconstituted skin using iPSC-derived fibroblasts. Red: keratin 14. Green: human type VII collagen (not mouse). Blue: DAPI. (D) Keratin 1 is present only in the suprabasal layer of epidermis. Green: human keratin 1 (does not recognize mouse keratin 1). Blue: DAPI. (E) Loricrin, a terminal differentiation marker of keratinocytes, is observed in the upper layer of epidermis. Red: loricrin. Green: human nuclei. Blue: DAPI.

 We next performed skin reconstitution chamber assays to test the *in vivo* functional capacity of iPSC-derived fibroblasts. We found that iPSC-derived fibroblasts combined with normal human keratinocytes grafted in the chamber contributed to reconstitution of normal skin structures on the back of SCID mice, similar to normal human fibroblasts and keratinocytes (Figure 3). Skin appendages, such as hair follicles and glands, were not detected, and no cyst or tumor formation was observed in the grafted sites. In addition, we confirmed that type VII collagen was expressed at the BMZ of the reconstituted skin (Figure 3C). The epidermal differentiation markers, keratin 1 and loricrin, were also expressed in the upper layers of the epidermis (Figure 3D and 3E), indicating that keratinocytes grown together with on iPSC-derived fibroblasts can be functionally and terminally differentiated. These data suggest that iPSC-derived fibroblasts have the capacity to support keratinocytes to constitute a multilayered epidermal structure and BMZ in *in vivo* reconstituted skin. 

### Generation of 3D Skin Equivalents Using iPSC-Derived Keratinocytes and Fibroblasts

**Figure 4:**
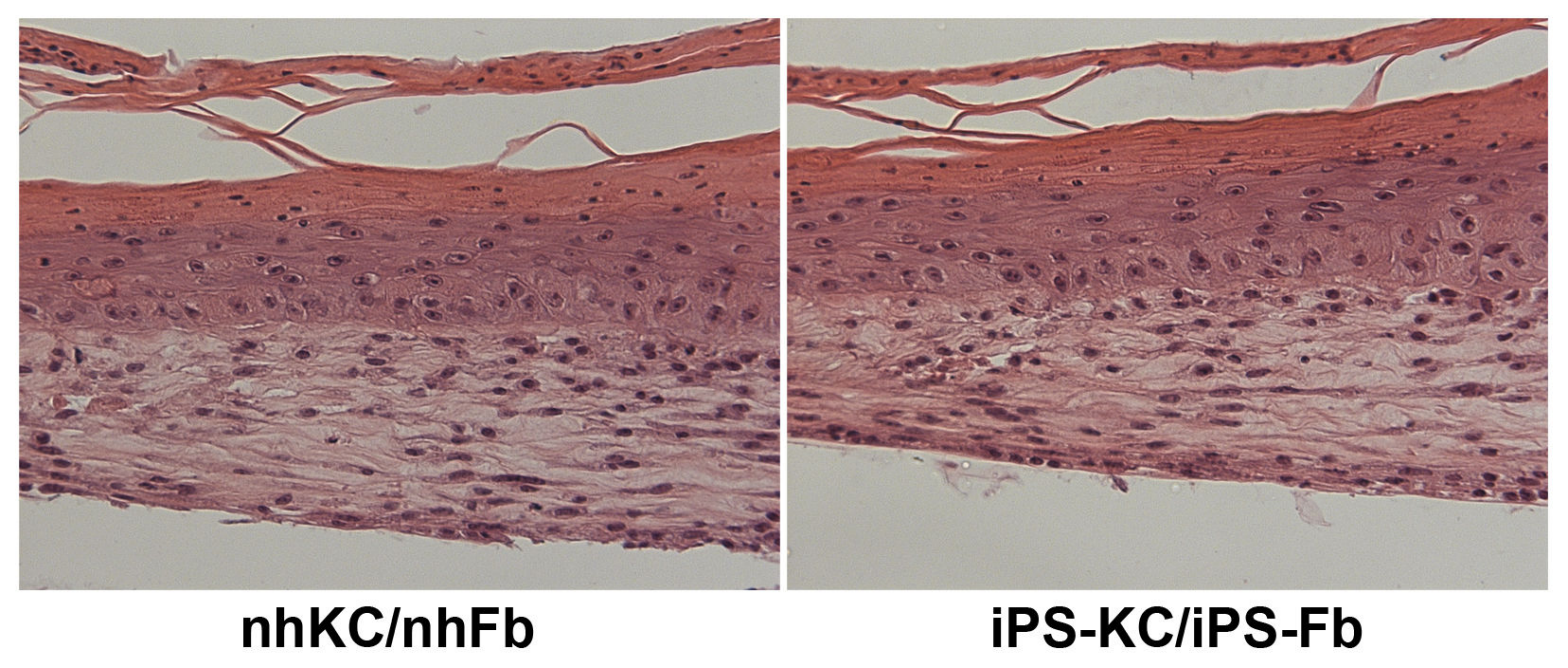
Generation of 3D skin equivalent. 3D skin equivalents were successfully generated using iPSC-derived keratinocytes and fibroblasts, and are histologically comparable to those generated using normal human keratinocytes and fibroblasts. H & E staining revealed normal epidermal and dermal morphology, as well as stratification and terminal differentiation.

 As a final step toward the future clinical use of iPSC-derived keratinocytes and fibroblasts, we next generated *in vitro* 3D skin equivalents using iPSC-derived cells in both components. iPSC-derived keratinocytes formed a multilayered epidermis and cornified layer at the surface of the epidermis on a dermis consisting of iPSC-derived fibroblasts, similar to those using normal human keratinocytes and fibroblasts (Figure 4). The presence of parakeratosis and the lack of a granular layer most likely occur as a general finding during the generation of 3D skin, since this was observed in both samples. These data further support the hypothesis that iPSC-derived fibroblasts can support the process of keratinocyte stratification, and that iPSCs have the capability to independently provide full-thickness artificial skin which contain both an epidermis and a dermis. 

## Discussion

 The discovery of iPSCs has had a profound impact on the field of stem cell research and regenerative medicine. In particular, the generation of PS-iPSCs is expected to provide an autologous source of cells to develop patient-specific customized cell and gene therapy [2], in large part by coupling this approach with gene correction. The methodology for correcting gene mutations in PS-iPSCs and directly differentiating iPSCs into skin cells is a critical step toward enabling the development of iPSC-based cell and gene therapy for skin diseases. The capability to correct pathogenic mutations in PS-iPSCs represents a critical advance for basic biomedical research and a significant step toward iPSC-based cell replacement therapies. 

Recent studies provided the first demonstrations of gene modification based on homologous recombination in ESCs and iPSCs using zinc-finger nucleases (ZFNs), a novel tool for gene modification which generates double-strand breaks at specific sites in the human genome [28]. Moreover, based on these studies, successful gene targeting has been reported in PS-iPSCs from some human monogenetic diseases [29–31]. For example, Yusa et al. showed that transduction of ZFNs in human iPSCs generated from an individual with α1-antitrypsin deficiency achieved correction of a point mutation in the α1-antitrypsin gene that underlies this deficiency. These authors also confirmed that genetic correction in human iPSCs restored the structure and function of α1-antitrypsin in subsequently derived liver-like cells [29], indicating that the combination of a genome editing technique such as ZFNs, and more recently transcription activator-like effector nucleases (TALENs) [32], together with iPSC technology can provide a generalizable approach to the treatment of monogenic diseases, including any of the types of EB. 

Although homologous recombination-based gene correction in PS-iPSCs from a skin disease has been not yet reported, there has recently been progress made in differentiation of iPSCs into skin cells. For example, our group previously demonstrated that human iPSCs could be differentiated into epidermal keratinocytes, one of main components of the skin [11]. We confirmed the functionality of iPSC-derived keratinocytes by assessing contribution to *in vitro* 3D skin formation and terminal differentiation capability in 3D skin models [11]. These studies demonstrated crucial proof-of-principle establishing the potential of iPSCs to generate autologous donor cells and subsequently keratinocytes for cell-based therapies for skin diseases. 

Here, we report the direct differentiation capability of iPSCs into fibroblasts, the second predominant cellular component of the skin. The cells have the capability to treat individuals with RDEB via their production of type VII collagen, and are also able to reconstitute full-thickness skin for regenerative therapy. In addition, fibroblasts may have another role in patients with RDEB in addition to merely supporting epidermal structure from the dermis. Although the mechanisms leading to the development of aggressive SSCs in RDEB patients is not well understood, loss of type VII collagen at the BMZ has been reported to increase the invasive behavior of RDEB-related SCCs [33]. A recent study showed that type VII collagen controls the expression level of dermal extracellular matrices (ECMs) derived from fibroblasts, not from keratinocytes [34,35], and that the reduction in these ECMs promotes RDEB-related SCC keratinocyte invasion. Moreover, these authors demonstrated that re-expression of type VII collagen in RDEB fibroblasts can reduce tumor cell invasion in 3D skin culture and restrict tumor growth *in vivo* [35], indicating that fibroblasts may potentially be more valuable than keratinocytes as a potential cell source for RDEB treatment, both to suppress SCC metastasis, as well as restore BMZ integrity. 

Previous studies have reported generation of fibroblasts from ESCs/iPSCs [36–38], however, these original protocols were not optimized specifically for generation of fibroblasts from ESCs/iPSCs. Instead, these methods simultaneously differentiated ESCs/iPSCs into a heterogeneous mix of differentiated cell populations, including both fibroblasts and epithelial cells, because of the use of keratinocyte medium and feeder cells which are required for keratinocyte culture, as well as the addition of bone morphogenetic protein 4 which is used for generating keratinocytes and other epithelial cells from ESCs/iPSCs [39,40]. Moreover, production of type VII collagen from fibroblasts derived by these protocols was not previously demonstrated [38]. 

Here, we focused on developing a method of specifically deriving fibroblasts from human iPSCs. Our protocol advances the generation of fibroblasts for future clinical use, because it can directly generate a relatively pure fibroblast population from iPSCs with high efficiency, based on cell surface marker analysis by FACS (Figure 3). There remains the possibility that this population may be heterogeneous, since it is difficult to completely define the characteristics of fibroblasts isolated from skin dermis, however, it is markedly enriched for fibroblasts. Additionally, our protocol is quite simple and we found that iPSC-derived fibroblasts generated in this manner can actively proliferate at least until passage 10. 

Type VII collagen matures via a polymerization process and the formation of tripeptide structures to generate collagen fibers. Therefore, using immunostaining and western blot analysis, we detected the tripeptide form of type VII collagen in medium cultured from fibroblasts derived from iPSCs by our protocol, demonstrating that our iPSC-derived fibroblasts had the capability to produce and secrete fully mature type VII collagen for the advancement of cell therapy for RDEB. 

We next interrogated the *in vivo* function of iPSC-derived fibroblasts. Skin reconstitution chamber assays clearly showed that iPSC-derived fibroblasts can reconstruct human skin structures *in vivo* with normal human keratinocytes (Figure 3). Moreover, positive staining of type VII collagen was observed at the BMZ of the reconstituted skin, indicating that iPSC-derived fibroblasts normally have two main *in vivo* functions of fibroblasts, not only to generate dermal structure and support keratinocytes to form a stratified epidermal structure, but also to form the BMZ along with keratinocytes. 

Taken together with our previous study for generating keratinocytes from iPSCs [11], we have succeeded in generating the two main cellular components of the skin. Moreover, we also developed an *in vitro* autologous basic 3D skin equivalent entirely reconstituted from iPSC-derived skin cells. To our knowledge, this is the first report to mimic a simple human organ *in vitro*, using two different lineages of cells derived from human iPSCs, albeit without skin appendages, vascularization or innervation. Our findings provide an innovate option for *in vitro* disease modeling and genetic and stem cell therapy for skin diseases, including RDEB, as well as different forms of EB and the other monogenic skin disorders. This approach will be particularly powerful when combined with methods to correct the underlying gene mutations in PS-iPSCs, and/or by generating PS-iPSCs from spontaneously gene-corrected revertant mosaic skin in which restoration of protein production has naturally occurred by spontaneous second-site mutations, which are observed in keratinocytes of patients with EB [41]. 

In conclusion, we report a new and efficient methodology for differentiating human iPSCs into fully functional dermal fibroblasts, which can synthesize mature type VII collagen to treat RDEB. Furthermore, we succeeded in generating full-thickness artificial skin using iPSC-derived keratinocytes and fibroblasts. Our study indicates that iPSCs have the potential to supply a source of both major types of skin cells, and produce an autologous 3D skin equivalent for generating *in vitro* skin disease models and developing gene and stem cell therapy for both inherited skin diseases, as well as more common applications such as postsurgical reconstruction and wound healing, among others. 

## Methods

### Cell Culture

We previously generated iPSC lines from human fibroblasts as previously described [11]. iPS1 and iPS2 in Figure 2 were different cell lines generated from fibroblasts isolated from a normal individual. iPSCs were maintained on a mitomycin C (MMC)-treated mouse embryonic fibroblast (MEF) feeder layer in medium for iPSC (iPSM: Knockout (KO)-DMEM supplemented with 20% KO-Serum Replacement, 1% GultaMax-I, 1% nonessential amino acid, 1% penicillin-streptomycin (Invitrogen) and 4ng/ml basic fibroblast growth factor (bFGF) (R&D systems). 

### iPSC Differentiation into Fibroblasts and Keratinocytes

In order to directly differentiate iPSCs into fibroblasts, we first generated embryoid bodies (cell aggregates of ESCs/iPSCs) [1] in iPSM without bFGF, and supplemented with 0.3 mM ascorbic acid (Sigma-Aldrich), 10ng/ml transforming growth factor beta2 (TGFβ2) (R&D systems) and ITS-A supplement (diluted as manufacturer's instruction) (Invitrogen) on a low-binding dish. For inducing cell outgrowth, embryoid bodies were attached to a gelatin-coated dish, and cultured in DMEM (with high glucose) (Invitrogen) supplemented with ascorbic acid and 20% bovine serum for 10 days. Cells that grew out from embryoid bodies were passaged and cultured every week to obtain consistent spindle-shaped cells (Figure 1A and C). 

iPSC-derived keratinocytes used in this study were generated by a modification of our protocol as previously described [11]. Briefly, small clumps of iPSCs were subcultured on Matrigel (BD Biosciences) in MEF-conditioned iPSM for 1 day. iPSCs were incubated in defined keratinocyte serum-free medium (DKSFM; Invitrogen) supplemented with 1 μM all-trans retinoic acid (Sigma) and 10 ng/mL bone morphogenetic protein 4 (BMP4; R&D Systems) for 4 days. Medium was changed to CnT-07 (CELLnTECH), and differentiated iPSCs were maintained in culture until 30 days for expansion and maintenance of iPSC-derived keratinocytes. 

### RT-PCR and PCR

RNA was extracted using the RNeasy Mini kit (Qiagen) and DNA was removed by DNase treatment (Invitrogen) to avoid genomic DNA amplification. Complementary DNA was synthesized using total 2µg RNA by Superscript III reverse transcriptase and Oligo(dT) primer (Invitrogen) according to the manufacturer's instructions. PCR reactions were performed with Platinum® PCR SuperMix (Invitrogen). All primer sequences are supplied in Table S1.

### FACS Analysis

The differentiated spindle-shape cells were enzymatically collected and fixed with 4% PFA/PBS for 5 minutes at RT. After PBS wash, cells were blocked in PBS with 10% goat serum. Primary antibodies conjugated with fluorescent molecules (Table S2) were incubated for 1 hour in PBS with 1% goat serum. Normal IgG and no primary antibody were used as negative controls. Stained cells were analyzed on BD LSRII Cell Analyzer using BD FACSDiva software (BD Biosciences) in order to calculate the differentiation efficiency of iPSCs into fibroblasts, and to determine the surface characteristics of iPSC-derived fibroblasts (n=3). 

### Immunofluorescence Staining

iPSC-derived fibroblasts were fixed with acetone/ethanol. After one hour of blocking using 10% goat or donkey serum in PBS, samples were incubated for one hour at room temperature (RT) or overnight at 4°C with primary antibodies. After three rinses with PBS, incubation with appropriate secondary antibodies was performed for 1 hour at RT. To detect human nuclear staining, frozen sections were fixed with 4% PFA in PBS, and performed blocking process using the M.O.M. immunodetection kit (Vector Laboratories) according to the manufacturer's instructions. Human nuclear antibody and secondary antibody were diluted with M.O.M. diluent and applied. Nuclear staining was performed by Vectashield®, mounting medium containing DAPI (Vector Laboratories). Confocal microscopy (Carl Zeiss LSM 5 Exciter) was used to visualize and capture immunostained cells with good resolution. All antibodies are supplied in Table S2.

### Western Blot Analysis

 In order to collect secreted type VII collagen from FBs and iPSC-FBs, subconfluent cell cultures were fed for 48 hours with serum-free medium supplemented with 50 ug/ml ascorbic acid. For SDS-PAGE analysis, the culture medium was treated with Amicon Ultra-100,000 Centrifugal Filter Devices (Millipore) for concentration and desalting. The samples were separated on a 5% polyacrylamide gel under reducing conditions. Immunoblotting analysis was performed using a rabbit polyclonal antibody against Collagen Type VII (Calbiochem) at 1:5,000 dilution. After overnight incubation at 4 degrees, the blot was washed with PBS with Tween 20 for 4 times, 15 min each at RT and then incubated with the goat anti-rabbit secondary antibody (1:10,000) and detected with the SuperSignal West Dura substrate following the manufacturer's instructions (Thermo Scientific).

### Skin Reconstitution Chamber Assay

 A sterile silicone chamber (Renner GmbH, Germany) was implanted into the back of SCID mice under anesthesia to prevent inward migration of cells from the adjacent skin. Ten million cells were prepared for each epidermal component (normal human keratinocytes) and dermal component (normal human fibroblasts or iPSC-derived fibroblasts), and mixed together followed by centrifugation. The resultant cell pellet, comprised of epidermal and dermal cells, was resuspended in PBS and deposited into the chamber. One week after placing cells, the silicone chamber was removed. Skin was harvested from the chamber sites 21 days later to observe reconstituted skin structures (n=2: In the first experiment, the mouse skin cells migrated over the grafted area, and overgrew. The results of the second experiment are shown.). 

### Generation of 3D Skin Equivalent

 3D skin equivalents were generated according to a previously described protocol [42]. Briefly, a type I collagen matrix (containing normal human fibroblasts isolated from foreskin, or iPSC-derived fibroblasts) was deposited onto polyethylene terephthalate membranes (BD Biosciences), and allowed to polymerize. After incubation of the polymerized matrix for 5–7 days, normal human keratinocytes or iPSC-derived keratinocytes were re-seeded on the matrix, and incubated for 6 days. The composite culture was raised to the air-liquid interface and fed from below to induce epidermal differentiation. 3D skin equivalents were harvested 7 days later (n=8).

## Supporting Information

Table S1 **List of primers used in this study.**
(DOCX)

Table S2 **List of antibodies used in this study.**
(DOCX)

## References

[1] Takahashi K, Tanabe K, Ohnuki M, Narita M, Ichisaka T et al. (2007) Induction of pluripotent stem cells from adult human fibroblasts by defined factors. Cell 131: 861–872. doi:10.1016/j.cell.2007.11.019. PubMed: 18035408. 18035408

[2] Onder TT, Daley GQ (2012) New lessons learned from disease modeling with induced pluripotent stem cells. Curr Opin Genet Dev 22: 500–508. doi:10.1016/j.gde.2012.05.005. PubMed: 22749051. 22749051 PMC3489983

[3] Uitto J, Christiano AM (1992) Molecular Genetics of the Cutaneous Basement Membrane Zone. J Clin Invest 90: 687–692. doi:10.1172/JCI115938. PubMed: 1381721. 1381721 PMC329917

[4] Fine J-D, Johnson LB, Weiner M, Li K-P, Suchindran C (2009) Epidermolysis bullosa and the risk of life-threatening cancers: the National EB Registry experience, 1986-2006. J Am Acad Dermat 60: 203–211. doi:10.1016/j.jaad.2008.09.035. PubMed: 19026465. 19026465

[5] Uitto J, Christiano AM, Mclean WHI, Mcgrath JA (2011) Novel Molecular Therapies for Heritable Skin Disorders. J Invest Dermatol 132: 820–828. PubMed: 22158553. 22158553 10.1038/jid.2011.389 PMC3572786

[6] Woodley DT, Krueger GG, Jorgensen CM, Fairley JA, Atha T et al. (2003) Normal and gene-corrected dystrophic epidermolysis bullosa fibroblasts alone can produce type VII collagen at the basement membrane zone. J Invest Dermatol 121: 1021–1028. doi:10.1046/j.1523-1747.2003.12571.x. PubMed: 14708601. 14708601

[7] Ortiz-Urda S, Lin Q (2003) Injection of genetically engineered fibroblasts corrects regenerated human epidermolysis bullosa skin tissue. J Clin Invest 111: 251–255. doi:10.1172/JCI200317193. PubMed: 12531881. 12531881 PMC151880

[8] Woodley DT, Remington J, Huang Y, Hou Y, Li W et al. (2007) Intravenously injected human fibroblasts home to skin wounds, deliver type VII collagen, and promote wound healing. Molecular Therapy 15: 628–635. doi:10.1038/sj.mt.6300041. PubMed: 17245357. 17245357

[9] Fritsch A, Loeckermann S (2008) A hypomorphic mouse model of dystrophic epidermolysis bullosa reveals mechanisms of disease and response to fibroblast therapy. J Clin Invest 118: 1669-1679. doi:10.1172/JCI34292. PubMed: 18382769. 18382769 PMC2276400

[10] Wong T, Gammon L, Liu L, Mellerio JE, Dopping-Hepenstal PJC et al. (2008) Potential of fibroblast cell therapy for recessive dystrophic epidermolysis bullosa. J Invest Dermatol 128: 2179–2189. doi:10.1038/jid.2008.78. PubMed: 18385758. 18385758

[11] Itoh M, Kiuru M, Cairo MS, Christiano AM (2011) Generation of keratinocytes from normal and recessive dystrophic epidermolysis bullosa-induced pluripotent stem cells. Proc Natl Acad Sci U S A 108: 8797–8802. doi:10.1073/pnas.1100332108. PubMed: 21555586. 21555586 PMC3102348

[12] Keller G (2005) Embryonic stem cell differentiation: emergence of a new era in biology and medicine. Genes Dev 19: 1129–1155. doi:10.1101/gad.1303605. PubMed: 15905405. 15905405

[13] Franceschi RT (1992) The role of ascorbic acid in mesenchymal differentiation. Nutr Rev 50: 65–70. PubMed: 1565288. 1565288 10.1111/j.1753-4887.1992.tb01271.x

[14] Pinnell S, Murad S, Darr D (1987) Induction of collagen synthesis by ascorbic acid: a possible mechanism. Arch Dermatol 123: 1684–1686. doi:10.1001/archderm.1987.01660360122023. PubMed: 2825607. 2825607

[15] Takahashi T, Lord B, Schulze PC, Fryer RM, Sarang SS et al. (2003) Ascorbic acid enhances differentiation of embryonic stem cells into cardiac myocytes. Circulation 107: 1912–1916. doi:10.1161/01.CIR.0000064899.53876.A3. PubMed: 12668514. 12668514

[16] Sharon N, Benvenisty N (2007) MESODERMAL DIFFERENTIATION. Human cell culture 6. Netherlands: Springer Verlag pp. 129–148.

[17] Lorda-Diez CI, Montero JA, Garcia-Porrero JA, Hurle JM (2010) Tgfbeta2 and 3 are coexpressed with their extracellular regulator Ltbp1 in the early limb bud and modulate mesodermal outgrowth and BMP signaling in chicken embryos. BMC Dev Biol 10: 69. doi:10.1186/1471-213X-10-69. PubMed: 20565961. 20565961 PMC2906442

[18] Singla DK, Kumar D, Sun B (2005) Transforming growth factor-beta2 enhances differentiation of cardiac myocytes from embryonic stem cells. Biochem Biophys Res Commun 332: 135–141. doi:10.1016/j.bbrc.2005.04.098. PubMed: 15896309. 15896309

[19] Watabe T, Miyazono K (2009) Roles of TGF-beta family signaling in stem cell renewal and differentiation. Cell Res 19: 103–115. doi:10.1038/cr.2008.323. PubMed: 19114993. 19114993

[20] Rudnicka L, Varga J (1994) Elevated expression of type VII collagen in the skin of patients with systemic sclerosis. Regulation by transforming growth factor-beta. J Clin Invest 93: 1709–1715. doi:10.1172/JCI117154. PubMed: 7512991. 7512991 PMC294224

[21] Mauviel A, Lapière JC, Halcin C (1994) Differential cytokine regulation of type I and type VII collagen gene expression in cultured human dermal fibroblasts. J Biol Chemistry 269: 25–28. PubMed: 8276802. 8276802

[22] Do T-V, Kubba LA, Du H, Sturgis CD, Woodruff TK (2008) Transforming growth factor-beta1, transforming growth factor-beta2, and transforming growth factor-beta3 enhance ovarian cancer metastatic potential by inducing a Smad3-dependent epithelial-to-mesenchymal transition. Mol Cancer Res 6: 695–705. doi:10.1158/1541-7786.MCR-07-0294. PubMed: 18505915. 18505915 PMC2927222

[23] Azhar M, Runyan RB, Gard C, Sanford LP, Miller ML et al. (2009) Ligand-specific function of transforming growth factor beta in epithelial-mesenchymal transition in heart development. Dev Dynam 238: 431–442. doi:10.1002/dvdy.21854. PubMed: 19161227. PMC2805850 19161227

[24] Togo S, Sato T, Sugiura H, Wang X, Basma H et al. (2011) Differentiation of embryonic stem cells into fibroblast-like cells in three-dimensional type I collagen gel cultures. In Vitro Cell Dev Biol Anim 47: 114–124. doi:10.1007/s11626-010-9367-2. PubMed: 21107747. 21107747 PMC3042114

[25] Chen H-F, Chuang C-Y, Shieh Y-K, Chang H-W, Ho H-N et al. (2009) Novel autogenic feeders derived from human embryonic stem cells (hESCs) support an undifferentiated status of hESCs in xeno-free culture conditions. Human Reproduction 24: 1114–1125. 19202140 10.1093/humrep/dep003

[26] Xu C, Jiang J, Sottile V, McWhir J, Lebkowski J et al. (2004) Immortalized fibroblast-like cells derived from human embryonic stem cells support undifferentiated cell growth. Stem Cells 22: 972–980. doi:10.1634/stemcells.22-6-972. PubMed: 15536188. 15536188

[27] Myllyharju J (2003) Prolyl 4-hydroxylases, the key enzymes of collagen biosynthesis. Matrix Biol 22: 15–24. doi:10.1016/S0945-053X(03)00006-4. PubMed: 12714038. 12714038

[28] Zou J, Maeder ML, Mali P, Pruett-Miller SM, Thibodeau-Beganny S et al. (2009) Gene targeting of a disease-related gene in human induced pluripotent stem and embryonic stem cells. Cell Stem Cell 5: 97–110. doi:10.1016/j.stem.2009.05.023. PubMed: 19540188. 19540188 PMC2720132

[29] Yusa K, Rashid ST, Strick-Marchand H, Varela I, Liu P-Q et al. (2011) Targeted gene correction of α1-antitrypsin deficiency in induced pluripotent stem cells. Nature 478: 391-394. doi:10.1038/nature10424. PubMed: 21993621. 21993621 PMC3198846

[30] Zou J, Mali P, Huang X, Dowey SN, Cheng L (2011) Site-specific gene correction of a point mutation in human iPS cells derived from an adult patient with sickle cell disease. Blood 118: 4599-4608. doi:10.1182/blood-2011-02-335554. PubMed: 21881051. 21881051 PMC3208277

[31] Howden SE, Gore A, Li Z, Fung H-L, Nisler BS et al. (2011) Genetic correction and analysis of induced pluripotent stem cells from a patient with gyrate atrophy. Proc Natl Acad Sci U S A 108: 6537–6542. doi:10.1073/pnas.1103388108. PubMed: 21464322. 21464322 PMC3080993

[32] Hockemeyer D, Wang H, Kiani S, Lai CS, Gao Q et al. (2011) Genetic engineering of human pluripotent cells using TALE nucleases. Nat Biotechnol 29: 731–734. doi:10.1038/nbt.1927. PubMed: 21738127. 21738127 PMC3152587

[33] Martins VL, Vyas JJ, Chen M, Purdie K, Mein CA et al. (2009) Increased invasive behaviour in cutaneous squamous cell carcinoma with loss of basement-membrane type VII collagen. J Cell Sci 122: 1788–1799. doi:10.1242/jcs.042895. PubMed: 19435799. 19435799 PMC2684833

[34] Watt SA, Pourreyron C, Purdie K, Hogan C, Cole CL et al. (2011) Integrative mRNA profiling comparing cultured primary cells with clinical samples reveals PLK1 and C20orf20 as therapeutic targets in cutaneous squamous cell carcinoma. Oncogene 30: 4666–4677. doi:10.1038/onc.2011.180. PubMed: 21602893. 21602893 PMC3219832

[35] Ng Y-Z, Pourreyron C, Salas-Alanis JC, Dayal JHS, Cepeda-Valdes R et al. (2012) Fibroblast-derived dermal matrix drives development of aggressive cutaneous squamous cell carcinoma in patients with recessive dystrophic epidermolysis bullosa. Cancer Res 72: 3522–3534. doi:10.1158/0008-5472.CAN-11-2996. PubMed: 22564523. 22564523

[36] Hewitt KJ, Shamis Y, Hayman RB, Margvelashvili M, Dong S et al. (2011) Epigenetic and Phenotypic Profile of Fibroblasts Derived from Induced Pluripotent Stem Cells. PLOS ONE 6: e17128. doi:10.1371/journal.pone.0017128. PubMed: 21386890. 21386890 PMC3046119

[37] Hewitt KJ, Shamis Y, Carlson MW, Aberdam E, Aberdam D et al. (2009) Three-dimensional epithelial tissues generated from human embryonic stem cells. Tissue Eng A 15: 3417–3426. doi:10.1089/ten.tea.2009.0060. PubMed: 19405784. PMC2792058 19405784

[38] Shamis Y, Hewitt KJ, Bear SE, Alt-Holland A, Qari H et al. (2012) iPSC-derived fibroblasts demonstrate augmented production and assembly of extracellular matrix proteins. In Vitro Cell Dev Biol Anim 48: 112–122. doi:10.1007/s11626-011-9478-4. PubMed: 22259014. 22259014

[39] Kawasaki H, Mizuseki K, Nishikawa S, Kaneko S, Kuwana Y et al. (2000) Induction of midbrain dopaminergic neurons from ES cells by stromal cell-derived inducing activity. Neuron 28: 31–40. doi:10.1016/S0896-6273(00)00083-0. PubMed: 11086981. 11086981

[40] Schuldiner M, Yanuka O, Itskovitz-Eldor J, Melton DA, Benvenisty N (2000) Effects of eight growth factors on the differentiation of cells derived from human embryonic stem cells. Proc Natl Acad Sci U S A 97: 11307–11312. doi:10.1073/pnas.97.21.11307. PubMed: 11027332. 11027332 PMC17196

[41] Jonkman MF (1999) Revertant mosaicism in human genetic disorders. Am J Med Genet 85: 361–364. doi:10.1002/(SICI)1096-8628(19990806)85:4. PubMed: 10398261. 10398261

[42] Carlson MW, Alt-holland A, Egles C, Garlick JA (2008) Three-Dimensional Tissue Models of Normal and Diseased Skin. Current Protoc Cell Biol 19: 1–23. PubMed: 19085986. 10.1002/0471143030.cb1909s41 PMC2811850 19085986

