# Supplementary material for: Generation of 3D Skin Equivalents Fully Reconstituted from Human Induced Pluripotent Stem Cells (iPSCs)
Source: PLoS One. 2013 Oct 11;8(10):e77673. doi: 10.1371/journal.pone.0077673 (PMC3795682; doi:10.1371/journal.pone.0077673)
Supplement: Table S1 — List of primers used in this study. (DOCX) [file pone.0077673.s001.docx]

| **Gene** | **Primer Sequence** | |
| --- | --- | --- |
| B2M | Forward | cccaagatagttaagtgggatcg |
|  | Reverse | aagcaagcaagcagaatttgg |
| endo-SOX2 | Forward | acaccaatcccatccacact |
|  | Reverse | gcaaacttcctgcaaagctc |
| endo-OCT4 | Forward | gtactcctcggtccctttcc |
|  | Reverse | caaaaaccctggcacaaact |
| CD10 | Forward | gcaagtggcgaagcttgaccg |
|  | Reverse | aggaccgagaggctgatctccagt |
| CD73 | Forward | tggctcctctcaatcatgccgct |
|  | Reverse | ggcgaccggataccacctcca |
| COL1A1 | Forward | gtgctcgtggaaatgatggt |
|  | Reverse | ctcctcgctttccttcctct |
| COL3A1 | Forward | gcaaagatggaaccagtgga |
|  | Reverse | aacaccaccacagcaaggac |

**Table S1.** List of primers used in this study.
