# Supplementary material for: Generation of 3D Skin Equivalents Fully Reconstituted from Human Induced Pluripotent Stem Cells (iPSCs)
Source: PLoS One. 2013 Oct 11;8(10):e77673. doi: 10.1371/journal.pone.0077673 (PMC3795682; doi:10.1371/journal.pone.0077673)
Supplement: Table S2 — List of antibodies used in this study. (DOCX) [file pone.0077673.s002.docx]

| **Primary antibodies** | | |
| --- | --- | --- |
| **Antibodies** | **Dilution** | **Company** |
| CD10-FITC |  | BD Biosciences |
| CD44-FITC |  | BD Biosciences |
| CD73-FITC |  | BD Biosciences |
| CD90-FITC |  | BD Biosciences |
| Anti-Collagen I | 1:500 | Abcam |
| Collagen Type III | 1:100 | Fitzgerald Industries |
| Anti-Collagen Type IV  α 2 Chain | 1:500 | Chemicon |
| Monoclonal anti-collagen  type VII | 1:500 | Sigma-Aldrich  (Immunostaining) |
| Vimentin (C-20) | 1:100 | Santa Cruz Biotechnology |
| P4HB | 1:300 | Novus Biologicals |
| Anti-Collagen Type VII Rabbit polyclonal antibody | 1:5000 | Calbiochem  (Western blot analysis) |
| Keratin 14 polyclonal antibody | 1:1000 | Covance |
| Mouse anti-human nuclei | 1:100 | Millipore |
| Mouse anti-Desmoglein-3 | 1:100 | Santa Cruz Biotechnology |
| Keratin 1 polyclonal antibody | 1:2000 | Covance |
| Loricrin polyclonal antibody | 1:500 | Covance |
| **Secondary antibodies** | | |
| Alexa Fluor 594 goat anti-rabbit IgG (H+L) | 1:1000 | Invitrogen |
| Alexa Fluor 488 goat anti-mouse IgG (H+L) | 1:1000 | Invitrogen |

**Table S2.** List of antibodies used in this study.
